# First Report of *Fusarium avenaceum* Causing Blight on *Juniperus formosana* in China: Morphological and Molecular Characterization

**DOI:** 10.3390/biology14040385

**Published:** 2025-04-07

**Authors:** Guohui Zhang, Huanxian Gu, Shengying Song, Qin Yang

**Affiliations:** 1 School of Life and Health Science College, Kaili University, Kaili 556011, China; yangqin1028518@126.com; 2 Forest Plant Quarantine Station of Kaili City, Kaili 556000, China; guhuanxian@163.com; 3 Qiandongnan Prefecture Institute of Forestry Science, Kaili 556000, China; qdnssy@126.com

**Keywords:** *Fusarium avenaceum*, blight, pathogen, *Juniperus formosana*

## Abstract

In October 2024, a large-scale blight was found on tender shoots (young green shoots) of *Juniperus formosana* in Kaili City, Guizhou Province, China. The pathogen was isolated and purified from the diseased tissues by the tissue isolation technique. Through ITS identification and microscopic examination, the pathogen was determined to be *Fusarium avenaceum*. This is the first discovery in China that the blight on *Juniperus formosana* is caused by *Fusarium avenaceum.*

## Simple Summary

*Juniperus formosana* is a significant garden plant. However, the occurrence of blight causes severe withering of the tender shoots, thereby diminishing its landscape ornamental value. This study aims to identify the type of disease currently affecting Juniperus formosana and the associated pathogenic fungi, providing a scientific basis for effective disease prevention and control.

## 1. Introduction

Blight poses a significant threat to plants of the Juniperus genus, with its pathogenicity and severity clearly demonstrated in previous studies [1,2,3]. When the disease reaches an advanced stage, *Juniperus formosana* exhibits pronounced symptoms of poor growth. These include slow growth, a reduction in the sprouting of new shoots, a sparse crown, and weakened tree vigor. Ultimately, *J. formosana* may wither and die. The finding that pathogens such as *Diplodia cupressi* cause blight on *Leyland cypress* was reported in 2015 [1], along with *Diplodia sapinea* causing diplodia tip blight on *Juniperus communis* [2] and *Alternaria* spp. and *Aspergillus* spp. causing *Juniperus monosperma* (Engelm.) Sarg. blight in Mexico [3]. These pathogens are highly pathogenic and can invade the tissues of juniper, triggering pathological changes. *Fusarium avenaceum* has been reported as the causal agent of shoot dieback in *Pinus mugo* [4], as well as postharvest fruit rot in pear (*Pyrus* spp.) [5,6].

At present, there are no research reports on the blight of *J. formosana*. In October 2024, blight was observed on *J. formosana* in Kaili City, Guizhou Province, China (Latitude: 26.563755, Longitude: 107.976475, Altitude: 822.29). Most *J. formosana* within the area of approximately 11,755 square meters in Jinquanhu Martyrs’ Cemetery in Kaili City has been afflicted by blight, causing huge losses to the local landscape industry. To identify the pathogenic fungi affecting *J. formosana* in China, this study carried out pathogen isolation, microscopic examination, and identification, aiming to provide scientific support for subsequent disease prevention and control efforts.

## 2. Materials and Methods

### 2.1. Disease Specimen Collection

In October 2024, a large-scale blight was found on *J. formosana* in Kaili City, Guizhou Province, China. Several diseased plants were randomly selected in Jinquanhu Martyrs’ Cemetery. Observations and descriptions were recorded of the morphology, color, and severity of damage on diseased branches of *J. formosana*. Photographs documented the affected plant parts. The occurrence of disease symptoms was recorded. Fresh specimens were collected and transported to the Plant Pathology Laboratory of Kaili University for pathogen isolation and identification.

### 2.2. Isolation, Purification, and Identification of Pathogens

The experimental culture medium used was PSA medium, prepared as follows: peel 200 g of potatoes, slice them, and boil in water for 20 min. Filter the mixture and adjust the filtrate volume to 1000 mL. Add 17 g of sucrose and 17 g of agar, heat to dissolve, and sterilize at high temperature.

The tissue isolation technique was applied to isolate pathogens from the diseased branches of *J. formosana*. Ten diseased trees were sampled for pathogen isolation. Briefly, three sections of infected tissue were excised using sterile scissors and sequentially disinfected by immersion in a 0.1% mercuric chloride solution for 2–3 min, followed by a brief 2–3 s rinse in 75% ethanol. Using sterile forceps, the tissue segments were arranged in a triangular pattern on potato sucrose agar (PSA) solid medium. The plates were then incubated in an inverted orientation at 25 °C in a controlled temperature incubator for 5–7 days.

To obtain pure cultures, the resulting colonies were purified twice using a sterile inoculation needle. From these purified colonies, a central portion of mycelium was carefully transferred onto a glass slide. For the measurement of the mycelium, the widest and the narrowest parts should be measured. The morphological characteristics of the pathogen were examined and documented under a microscope, with measurements of mycelial and spore dimensions recorded (50 spores).

For molecular characterization, DNA sequencing targeting the ribosomal rDNA-ITS (Internal Transcribed Spacer) region was performed. The obtained ITS sequences were aligned and compared with reference sequences in the GenBank database using the online version of BLAST analysis (National Institutes of Health (NIH), Bethesda, MD, USA, https://blast.ncbi.nlm.nih.gov/, accessed on 28 November 2024). The pathogen was ultimately identified and classified by integrating molecular data with morphological observations, supported by the construction of phylogenetic dendrograms. Genomic DNA was extracted using the Ezup Column Fungal Genomic DNA Extraction Kit (Sangon Biotech, Shanghai, China) according to the manufacturer’s instructions. The internal transcribed spacer (ITS) region was amplified using the primers ITS1 (TCCGTAGGTGAACCTGCGG) and ITS4-R (TCCTCCGCTTATTGATATGC). The PCR products were sequenced, and the resulting sequences were used for phylogenetic tree construction.

After sequencing the ITS region of the unknown fungal isolate, BLAST analysis against the NCBI database revealed a 100% sequence similarity with the ITS sequence of *Fusarium avenaceum*, indicating that the isolate is likely related to *F. avenaceum*. To confirm this identification, additional ITS sequences from other Fusarium species were downloaded from the NCBI database. All sequences were aligned using the ClustalW function in MEGA 5 software (Tempe, AZ, USA), and alignment edges were trimmed to ensure a uniform sequence length. Subsequently, a phylogenetic tree was constructed based on the aligned sequences using the Neighbor-Joining (NJ) method implemented in MEGA 5 software. The reliability of the inferred phylogeny was tested using the bootstrap method, with 1000 replicates.

### 2.3. Pathogenicity Test

The purified pathogenic fungi were initially cultured on potato sucrose agar (PSA) medium under aseptic conditions and incubated at 25 °C for 5–7 days to induce sporulation. Following incubation, a spore suspension was prepared by gently dislodging fungal spores from the surface of the PSA medium using sterile distilled water. The spore concentration was standardized to 10^6^ spores/mL using a hemocytometer to ensure consistency and reproducibility during inoculation. For field inoculation, the spore suspension was applied to *J. formosana* branches using the needle puncture method. In this approach, small wounds were created on the branches with a sterile needle, and 10–50 µL of the spore suspension was directly introduced to each wound site. Control plants were treated with sterile distilled water using the same procedure. The inoculation site was wrapped with a sealing film. Pathogen inoculation was carried out on the tender (or young) shoots of branch tissues. We inoculated 10–20 branches, at 25–28 °C, and symptoms were observed 5 days later. Post-inoculation, the plants were monitored daily, and the development of lesions on branches was documented [6].

Once visible disease symptoms were observed, tissue samples were collected from the infected regions. These diseased tissues were excised using sterile scissors and surface-sterilized by sequential immersion in a 0.1% mercuric chloride solution for 2–3 min, followed by a brief 2–3 s rinse in 75% ethanol, and three subsequent rinses in sterile distilled water to eliminate surface contaminants. The sterilized tissues were then placed on PSA medium and incubated at 25 °C to facilitate fungal growth. The resulting fungal isolates were purified through single-spore isolation and subsequently identified by integrating morphological characterization with molecular analysis.

## 3. Results

### 3.1. Symptom Observation

**Figure 1:**
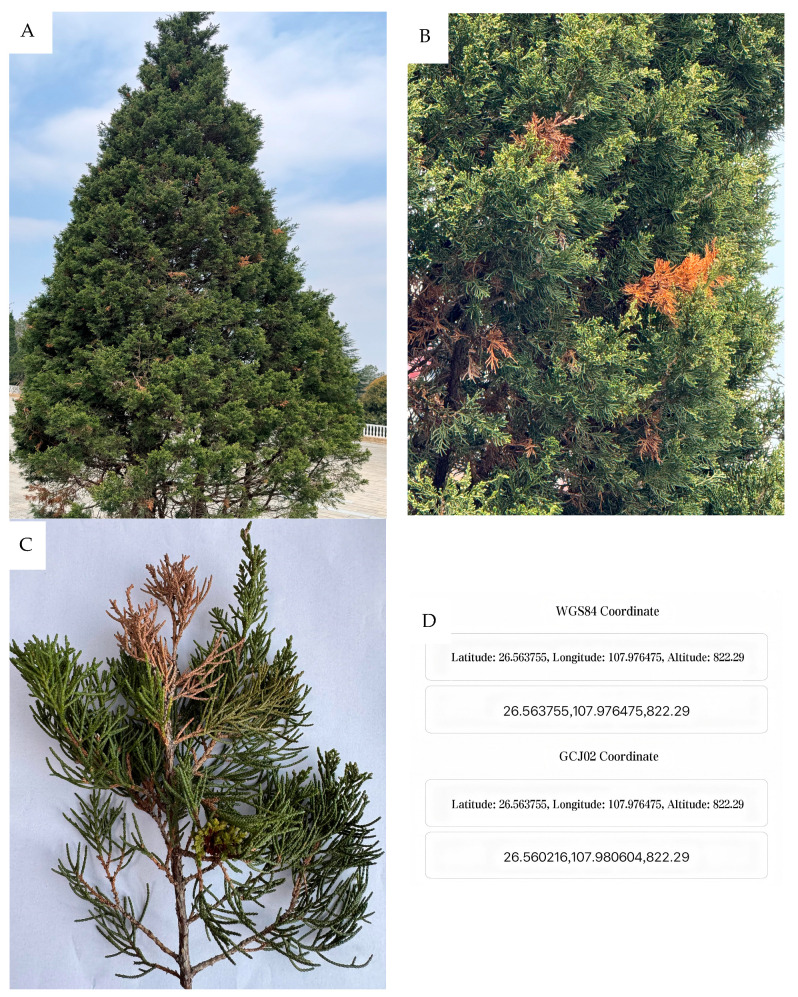
Symptoms of the blight disease on *Juniperus formosana*. The blight symptoms on *J. formosana* (**A**), the tips of the blight needles of *J. formosana* (**B**,**C**), and the GPS coordinates of the sampling site (**D**).

At the early stage of the disease, the shoot apexes of *J. formosana* branches exhibit chlorosis, with their color gradually changing from bright green to yellowish-green or grayish-green. In severe cases, most of the branches of *J. formosana* may be affected, resulting in a sparse canopy and weakened tree vigor (Figure 1A). The tips of the needles with tip branches (15–25 cm) begin to turn yellow and dry out, with the discoloration spreading toward the base, eventually causing the entire needle to turn brown or grayish-white. When the disease is severe, the affected branches gradually wither (Figure 1B,C, and the GPS coordinates of the sampling site are given in Figure 1D).

### 3.2. Identification of the Pathogen

**Figure 2:**
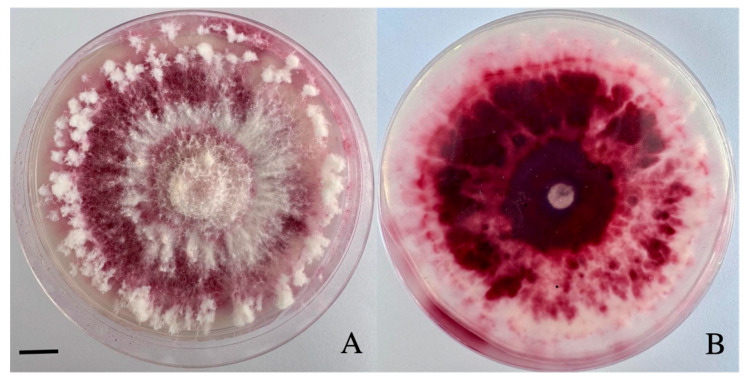
The colony morphology of the pathogen *Fusarium avenaceum.* Front view (**A**) and back view (**B**) of the colony (bar = 10 mm).

After 10 days, the mycelial colony on PSA was covered with tomentose aerial mycelia in white and red. The aerial mycelium grew vigorously. It was white, and it turned purple–red in the later stage (Figure 2A) as it contained purple–red pigments (Figure 2B).

**Figure 3:**
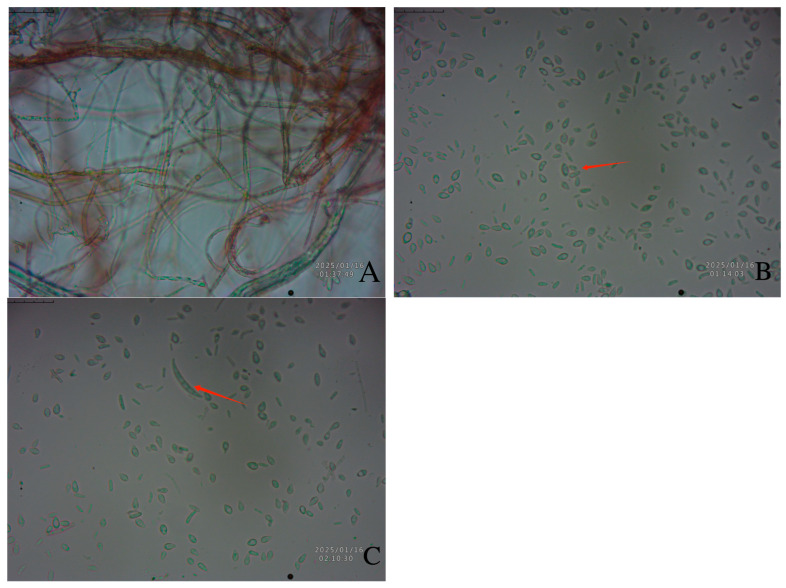
Morphological characteristics of *Fusarium avenaceum.* Mycelium (**A**), microspore (**B**), and macrospore (**C**) (10 × 40, bar = 10 μm).

**Figure 4:**
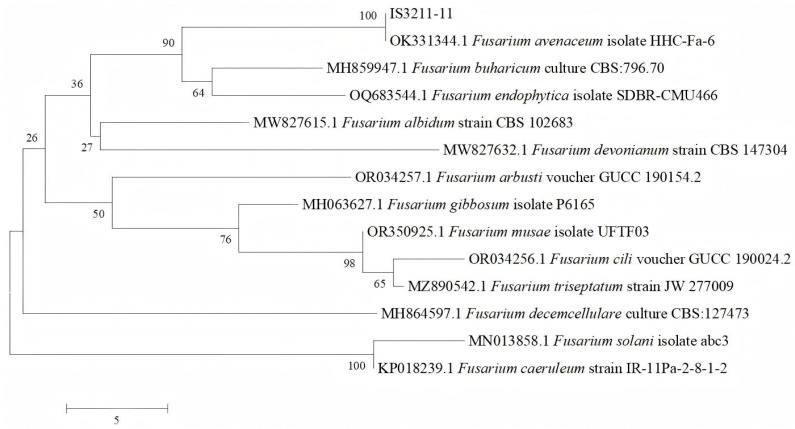
The dendrogram of *Fusarium avenaceum*.

On PSA, mycelia are colorless and transparent in the initial growth stage, turn red in the mature stage, and have septa (Figure 3A). The microconidia are elliptical or ovoid with 0–2 septa, measuring (3.05–18.80) μm × (1.75–2.39) μm (Figure 3B); the macroconidia are falcate, slender, usually have three to five septa, measure (24.05–53.80) μm × (3.35–6.09) µm, and are found in an extremely small number (Figure 3C). Based on morphological identification and ITS sequence analysis (primer sequences ITS1: TCCGTAGGTGAACCTGCGG; ITS4-R: TCCTCCGCTTATTGATATGC), we analyzed the pathogenic fungus (Table 1) using dendrograms (Figure 4), and the pathogen was identified as *Fusarium avenaceum*.

### 3.3. Pathogenicity Test and Isolation of the Fungus

**Figure 5:**
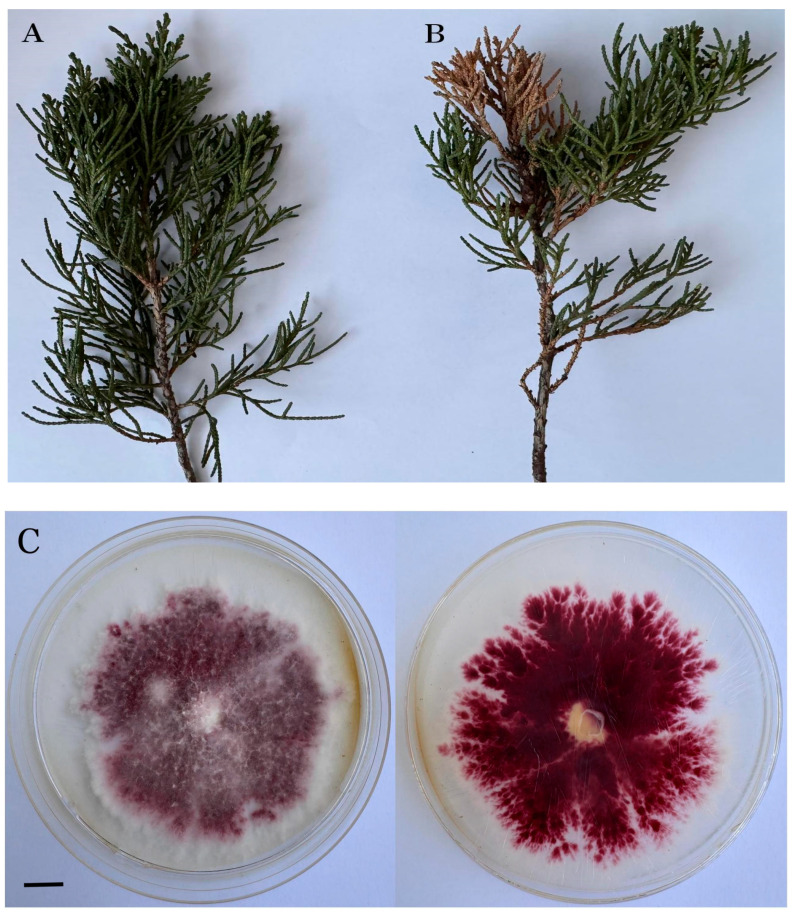
The pathogenicity test of *Fusarium avenaceum*. CK (**A**), the diseased needle (**B**), and the isolated strains (**C**) (bar = 10 mm).

To confirm the pathogenicity of the inoculated fungal strains on *J. formosana*, a spore suspension with a concentration of 10^6^ spores/mL was prepared and applied to the branches using the needle puncture method. Five to seven days post-inoculation, lesions were observed on the branches of the plant (Figure 5A,B), indicating infection by the pathogenic fungus (*Fusarium avenaceum*) (Figure 5C). Subsequently, these pathogenic fungi were isolated from the diseased tissues using tissue isolation techniques after seven days, and their identities were confirmed through microscopic analysis. The isolated strains were found to be identical to the initially inoculated strains. According to Koch’s postulates, these findings confirm that the inoculated strains are indeed the causative agents of the observed diseases in the branches of *J. formosana*.

## 4. Discussion

Needle blight is a plant disease characterized by needle wilting, discoloration, and lesion formation. In this study, a previously unreported needle blight was observed on *Juniperus formosana*, with symptomatic needles exhibiting yellowing and wilting along affected branches. Through a combination of morphological characterization and internal transcribed spacer (ITS) sequence analysis, and subsequent phylogenetic evaluation using a dendrogram, the causal pathogen was identified as *Fusarium avenaceum*. *Juniperus formosana* is a species of high ecological, ornamental, economic, and medicinal value, underscoring its importance as a multifunctional plant resource [7,8]. Despite its significance, current knowledge regarding diseases affecting *J. formosana* remains limited, and there is a lack of established scientific methods for disease prevention and control. This knowledge gap has contributed to the increasing severity of disease outbreaks, posing a serious threat to the sustainable and healthy development of *J. formosana* populations.

In this study, the morphological characteristics and phylogenetic relationships (as shown through a dendrogram) of the pathogenic fungus *Fusarium avenaceum* were analyzed from a professional plant pathological perspective. To date, there have been no published studies reporting blight disease in *Juniperus formosana* within Guizhou Province, China. This research provides a comprehensive description of the fungal pathogen, after employing both morphological (physical traits) and molecular (DNA-based) approaches to accurately identify and characterize the pathogen and elucidate its role in disease development [9]. Additionally, another report referenced in this study documents the first confirmed case of bulb rot caused by *F. avenaceum* in *Fritillaria taipaiensis* P. Li in China [10]; however, it lacks high-quality professional imagery to support the findings. Other existing studies on *F. avenaceum* are largely limited to the morphological and molecular characterization of Fusarium species isolates, without extensive pathological analysis or comprehensive documentation of host–pathogen interactions [11,12,13,14,15,16]. The morphological characteristics and dendrogram of the pathogenic fungus *F. avenaceum* were reported from a professional pathological perspective. *F. avenaceum* was described as causing root rot in pea [17,18,19], and all strains of *F. avenaceum* isolated from spring wheat and weeds were shown to have the potential to produce enniatins and moniliformin in spring wheat [20]. It also play a role in the decline of beech seedlings [21]. There are further reports in woody species of *F. avenaceum* causing shoot dieback of *Pinus mugo* [4] and postharvest decay of pear [5,6]. Research has demonstrated that p-hydroxybenzoic acid (pHBA) effectively inhibits the mycelial growth of *F. avenaceum* in a concentration-dependent manner. Specifically, pHBA at a concentration of 10 mM reduced mycelial growth by 59.8%. The half-maximal inhibitory concentration (IC_50_) of pHBA against *F. avenaceum* was determined to be 7.12 mM [22]. In comparison to carbendazim and tebuconazole, metconazole exhibited a superior antifungal efficacy. The effective concentration required to inhibit 50% of fungal growth (EC_50_) for metconazole was 0.56 mg/L against *F. avenaceum* [23].

## 5. Conclusions

Currently, there is no research available on blight in *J. formosana* in Guizhou Province, China. *F. avenaceum* was first identified in *J. formosana*, highlighting the importance of accurately identifying this pathogen. The focus of this study is on identifying pathogenic fungi affecting *J. formosana*, which holds substantial scientific significance for the cultivation and disease prevention of this species. By professionally determining the pathogenic fungi responsible for disease occurrence, this research adds to the list of known pathogens associated with *J. formosana,* laying a strong foundation for future in-depth studies.

## Figures and Tables

**Table 1:** IS3211 (ITS Sequences).

CCTGCGGAGGGATCATTACCGAGTTTACAACTCCCAAACCCCTGTGAACATACCTTAATGTT GCCTCGGCGGATCAGCCCGCGCCCCGTAAAACGGGACGGCCCGCCAGAGGACCCAAACTCTAATGTTTCTTA TTGTAACTTCTGAGTAAAACAAACAAATAAATCAAAACTTTCAACAACGGATCTCTT GGTTCTGGCATCGATGAAGAACGCAGCAAAATGCGATAAGTAATGTGAATTGCAGAATTCAGTGAATCATCG AATCTTTGAACGCACATTGCGCCCGCTGGTATTCCGGCGGG CATGCCTGTTCGAGCGTCATTTCAACCCTCAAGCCCCCGGGTTTGGTGTTGGGGATCGGCTCTGCCTTCTGGCG GTGCCGCCCCCGAAATACATTGGCGGTCTCGCTGCAGCCTCCATTGCGTAGTAGCTAACAC CTCGCAACTGGAACGCGGCGCGGCCATGCCGTAAAACCCCAACTTCTGAATGTTGACCTCGGATCAGGTAGG AATACCCGCTGAACTTAAGCATATC

## Data Availability

The original contributions presented in this study are included in the paper. Further inquiries can be directed to the corresponding author.
